# Classification of tomato leaf images for detection of plant disease using conformable polynomials image features

**DOI:** 10.1016/j.mex.2024.102844

**Published:** 2024-07-03

**Authors:** Ala'a R. Al-Shamasneh, Rabha W. Ibrahim

**Affiliations:** a Department of Computer Science, College of Computer & Information Sciences, Prince Sultan University, Rafha Street, Riyadh 11586, Saudi Arabia; b Faculty of Engineering and Natural Sciences, Advanced Computing Lab, Istanbul Okan University, 34959, Türkiye; c Information and Communication Technology Research Group, Scientific Research Center, Alayen University, Nile Street, 64001, Dhi Qar, Iraq

**Keywords:** Tomato disease detection, Feature extraction, Conformable polynomials, Classification, Classification of tomato leaf images for detection of tomato disease

## Abstract

Plant diseases can spread rapidly, leading to significant crop losses if not detected early. By accurately identifying diseased plants, farmers can target treatment only to the affected areas, reducing the number of pesticides or fungicides needed and minimizing environmental impact. Tomatoes are among the most significant and extensively consumed crops worldwide. The main factor affecting crop yield quantity and quality is leaf disease. Various diseases can affect tomato production, impacting both yield and quality. Automated classification of leaf images allows for the early identification of diseased plants, enabling prompt intervention and control measures. Many creative approaches to diagnosing and categorizing specific illnesses have been widely employed. The manual method is costly and labor-intensive. Without the assistance of an agricultural specialist, disease detection can be facilitated by image processing combined with machine learning algorithms. In this study, the diseases in tomato leaves will be detected using new feature extraction method using conformable polynomials image features for accurate solution and faster detection of plant diseases through a machine learning model. The methodology of this study based on:

• Preprocessing, feature extraction, dimension reduction and classification modules.
• Conformable polynomials method is used to extract the texture features which is passed classifier.
• The proposed texture feature is constructed by two parts the enhanced based term, and the texture detail part for textual analysis.
• The tomato leaf samples from the plant village image dataset were used to gather the data for this model. The disease detected are 98.80 % accurate for tomato leaf images using SVM classifier. In addition to lowering financial loss, the suggested feature extraction method can help manage plant diseases effectively, improving crop yield and food security.

## Graphical abstract

**Figure:**
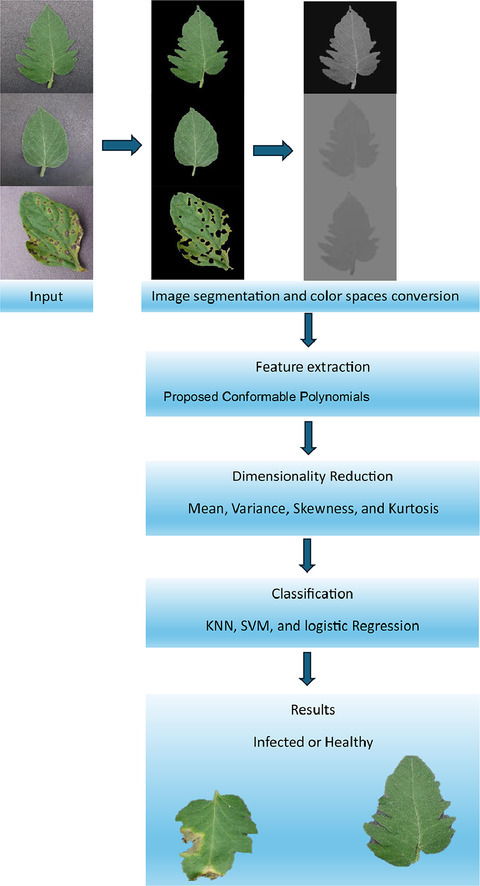

Specifications table

**Table:** 

Subject are:a	
More specific subject area:	Image processing
Name of your method:	Classification of tomato leaf images for detection of tomato disease
Name and reference of original method:	Not applicable
Resource availability:	Plant Village image dataset

## Background

Improving agricultural productivity can directly robust the economy and indirectly alleviate poverty with the growth in the agriculture sector. As a part of it the timely diagnosis of illnesses on plant leaves is the key concern. Large losses in the production of fresh and processed crops have been caused by a number of diseases that have threatened crop production. Numerous factors, including bacterial, viral, and fungal infections, contribute to the development of these diseases [1]. Warm temperatures and extended periods of moisture, which are common in most tomato-producing regions, are favorable conditions for the majority of foliar diseases, including late blight, target, and bacterial spots. One of the most important and widely consumed crops in the world is the tomato. Saudi Arabia is one of the largest agricultural markets in the region. The market is expanding primarily due to government initiatives aimed at reducing concerns about food security, the significant shift toward greenhouse production, and the growing demand for organic food products. Moreover, individuals are shifting to nutritional diet plans due to the rising incidence of lifestyle diseases and increasing health consciousness. For instance, tomato production in the country increased by 104.9 % between 2018 and 2022 [2].

Global agricultural productivity can indeed be adversely affected by various factors, including changing weather patterns, extreme temperatures, droughts, floods, and other climate-related events can disrupt agricultural activities, affecting crop yields and livestock production. The insects, fungi, weeds, and other pests can damage crops, leading to significant losses in agricultural productivity if not adequately managed. Moreover, the areas affected by wars and conflicts often experience disruptions in agricultural production due to destruction of infrastructure, displacement of farmers, and limited access to resources such as water, seeds, and fertilizers.

The most sensitive part of a plant is leaf, they are the first to display disease signs. A plant disease can be identified by looking for particular symptoms. Although dark patches or yellowing on leaves are early indicators of many infections, closer examination helps identify the underlying cause. Throughout their growth cycle, crops need to undergo regular inspections to detect any potential disease or illness. This process is necessary to ensure that the crops are healthy and free from any harmful agents before they are ready for harvest. Currently, the process of identifying crop diseases relies on manual methods carried out by farmers, which not only consumes valuable time but also introduces subjectivity and potential errors due to human involvement [3]. Fortunately, this issue can be addressed effectively by leveraging computer intelligence and image processing tools [4,5]. By utilizing these technologies, farmers can benefit from accurate and efficient diagnoses of crop diseases. Modern machine learning detection systems often pre-process images of infected leaves using low-level image processing methods, such as noise removal, semantic procedures, and image refinement [6,7]. Precision agriculture has automated the detection and identification of tomato leaf disease through the use of numerous artificial intelligence (AI) techniques. These techniques are motivated by the remarkable success of artificial intelligence (AI) technologies, such as the most recent deep learning (DL) techniques and the classic machine learning (ML) algorithms [8,9]. Plant disease classification tasks currently make extensive use of machine vision technology and deep learning (DL) algorithms, particularly those that are based on convolutional neural networks (CNNs) [10,11].

Several studies have been conducted to identify plant diseases, and a large part of these studies rely on the manually created features that are given to the classifiers in order to improve their accuracy [12]. Furthermore, scars on leaves are among the most noticeable signs of plant disease. In contrast to their healthy counterparts, the infected leaves are dispersed unevenly in terms of texture or color. Moreover, infected leaves have distinct disease spot shapes. Numerous researchers have looked into the different imaging techniques and approaches for extracting disease features. The leaf photos were taken using scientific techniques, and classification models were created [1,13]. Several approaches for tomato leaf disease classification have been proposed based on either image processing and machine learning or deep learning to construct automated disease detection systems.

The image processing and machine learning approaches involve preprocessing images of tomato leaves to extract relevant features such as texture, color, and shape. These features are then used as input to machine learning algorithms to classify the leaves into healthy or different disease categories. While the deep learning approaches are capable of automatically learning relevant features from raw image data without the need for explicit feature extraction. Existing literature mostly focuses on either handcrafted or deep learning-based methods in isolation. As mentioned, the handcrafted features capture domain-specific knowledge, while deep learning features discern complex, abstract patterns within the images. Therefore, this study aims to use the handcrafted features that are extracted from tomato leaves to provide additional diagnostic information for classifying tomato leaves as healthy or infected. This study's main contribution is the use of the proposed conformable polynomials model to capture the high frequency component of tomato leaf image details, which will improve classification accuracy.

Majority of leaf diseases are manifested in the form of small spots that may vary in size and appearance. Automated tomato leaf disease classification refers to the use of machine learning or artificial intelligence algorithms to identify and classify diseases affecting tomato plants based on images of their leaves. This technology can assist farmers and agricultural experts in early detection and management of diseases, thus potentially improving crop yield and reducing losses [14]. These days, a large area of research is focused on applications based on image processing for the identification and classification of plant diseases. Numerous scholars have examined diverse imaging techniques and methods for extracting disease features [1,10]. Color, shape, and texture features were extracted from images of healthy and unhealthy tomato plants for the study that proposed by Sabrol and Satish [15]. The feature extraction procedure is carried out following the segmentation step. A total of 97.3 % classification accuracy was obtained from the classification of six different tomato image types. Several approaches for tomato leaf disease classification have been proposed, the classification algorithm and fusion of multiple features for identifying and categorizing tomato leaf disease were introduced by Basavaiah and Arlene Anthony [16]. Color histograms, Local Binary Pattern, and Hu Moments features are used for training and testing, while decision tree classification and Rrandom forest are used for classifying leaf diseases. The decision tree classifier achieved 90 % classification accuracy, while the random forest classifier achieved 94 %. Badiger et al. [17], developed a leaf disease classifier using SVM. The authors standardized the image sizes and applied k-means clustering for image segmentation. The SVM classified diseases using GLCM features. Deshapande et al. [35], implemented a machine-learning algorithm for disease classification in maize leaves. The authors utilized eighteen histogram features and eight Haar wavelet features with SVM and KNN classifiers. These classifiers achieved an accuracy of 85 % for KNN and 88 % for SVM. In another study [36], researchers focused on classifying infected tobacco leaves with 120 leaf images. They implemented a CNN model and compared it with existing models, demonstrating an accuracy of 85.1 % for their proposed model.

The study by Lubis et al. [18] proposed the K-Nearest Neighbor (K-NN) algorithm and feature extraction for the classification of tomato leaf disease images. This study achieved classification accuracy of 96.58 % the KNN algorithm.

The study by Kiran and Chandrappa [19] developed an effective method for plant leaf disease detection using computer vision techniques. In this study the leaf disease detection comprised histogram equalization, and different feature descriptors. These features are then used to classify the images. The classification accuracy improved up to 97.92 % using the Random Forest classifier.

In computer vision, the deep learning (DL) has become a popular family of machine learning algorithms that has been effectively applied to a variety of problems. Due to its hierarchical technique of learning high-level from input data, the DL employing the CNN is quickly increasing in image classification. Several studies classified tomato leaf diseases using DL algorithms that made use of various CNN architectures [20].

The research by Trivedi et al. [11] used a deep neural network model to identify and categorize leaf diseases in tomato plants into pre-established groups. It also considered the morphological characteristics of the plant, including its color, texture, and leaf margins. This work presented standard models for profound learning along with their variations.

In their study, [12] suggested a pipeline that uses three compact CNNs to automatically identify tomato leaf diseases. Transfer learning is used to extract deep features for a more simplified representation. Additionally, the process for identifying tomato leaf illnesses used six classifiers. The results show that the K-NN and SVM classifiers have the highest accuracy. The large number of deep layers and numerous parameters in these CNN approaches necessitate a high computational capacity to update the hyperparameters, which increases the classification complexity. The lack of labelled data is one of the most critical challenges to deep learning in image applications. Deep learning models often require significant computational resources, especially during training. Handcrafted feature extraction can sometimes result in simpler models with lower computational requirements, making them more suitable for deployment in resource-constrained environments. Handcrafted features can sometimes be transferable across different datasets or tasks within the same domain. Once a set of effective features has been identified for tomato leaves classification, it may be possible to reuse or adapt them for related tasks, potentially saving time and effort in feature engineering. The primary contribution of this study is the developmental model for handcrafted extracting features from tomato leaves to achieve superior classification accuracy. By managing non-linearity and noise, reducing dimensionality, delivering a richer, more discriminative representation of image data, and ultimately enhancing the performance of classification algorithms, the conformable polynomials feature extraction method improves image classification. When images have intricate relationships and patterns that are difficult for conventional linear models to capture, this method is especially helpful.

## Method details

**Fig. 1:**
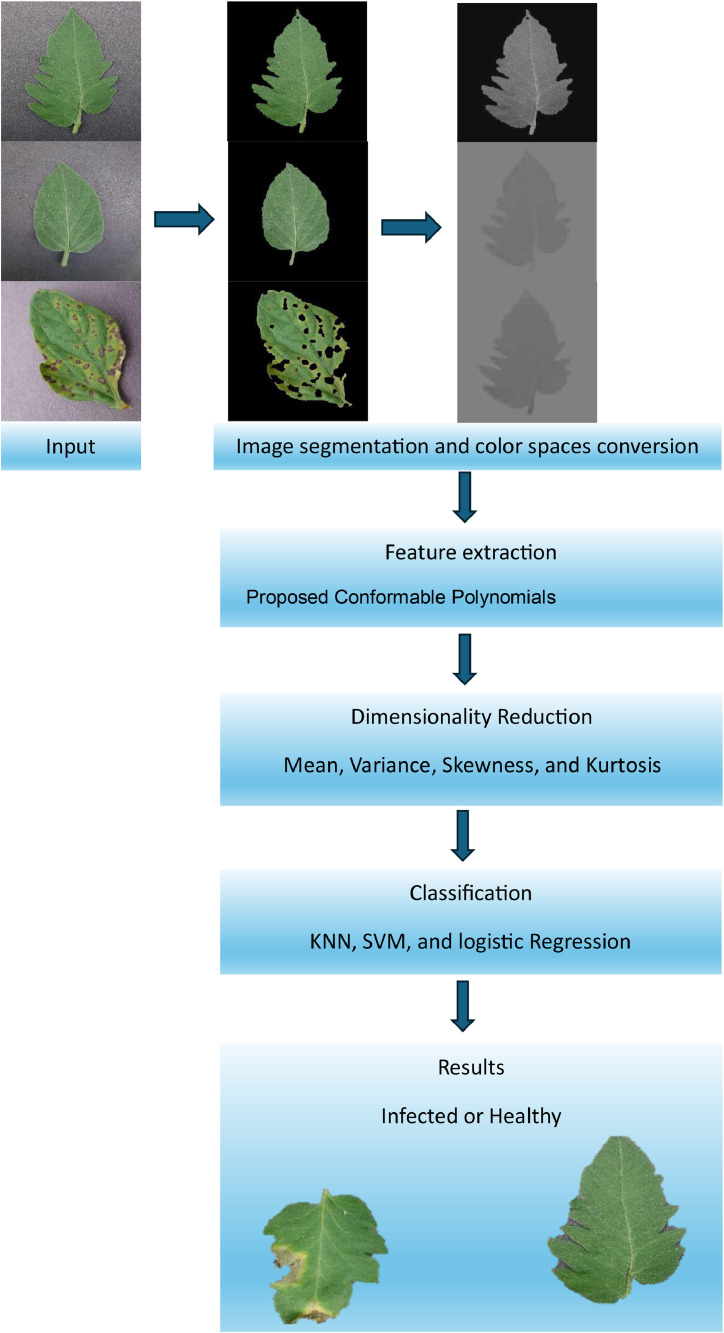
Block diagram of the proposed image preprocessing.

The traditional method of handcrafted techniques includes characteristics like texture and shape. After that, a machine learning algorithm receives these features as input. The manual feature extraction techniques rely on texture analysis to identify images of tomato leaves as either healthy or infected images. This study involves extracting features using conformable polynomials approach. The proposed study includes A. Data Acquisition; Pre-Processing; feature extraction using proposed CPs, dimension reduction, and finally classification. The flow process for classification is illustrated in Fig. 1.

## Image acquisition

**Fig. 2:**
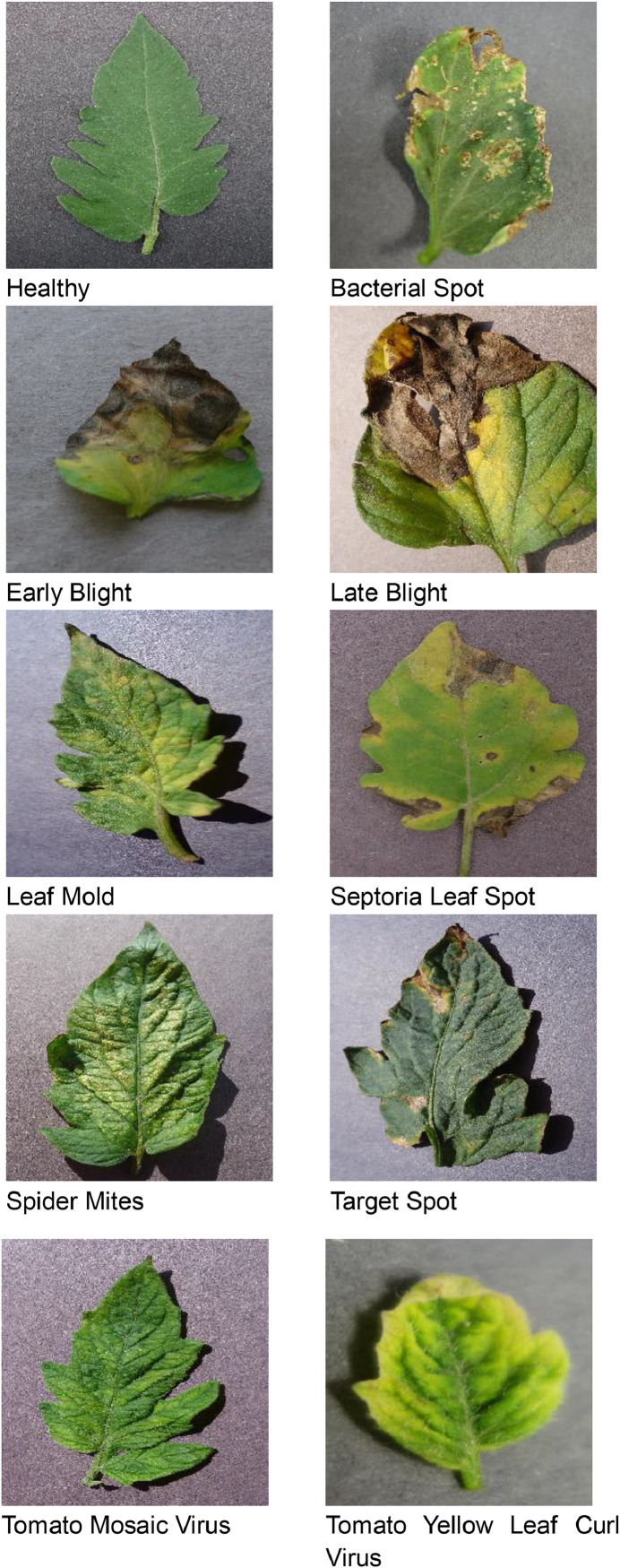
Examples of tomato from Plant Village dataset.

The data or images of tomato leaf infection have been collected from Plant Village repository [21]. More than 50,000 images of 14 different crops, including tomato, grape, apple, corn, and soybean, are included in the dataset. The collected dataset has almost 18,200 images that belongs to various classes. Sample of images are illustrated in Fig. 2.

## Image preprocessing

Pre-processing often involves transforming the data into a format that is more suitable for the classification algorithm. In this study the identifying and selecting the most relevant features can improve the performance of the classification model by reducing dimensionality and focusing on the most informative aspects of the data. The pre-processing of the image includes the following stapes.

## Image segmentation

**Fig. 3:**
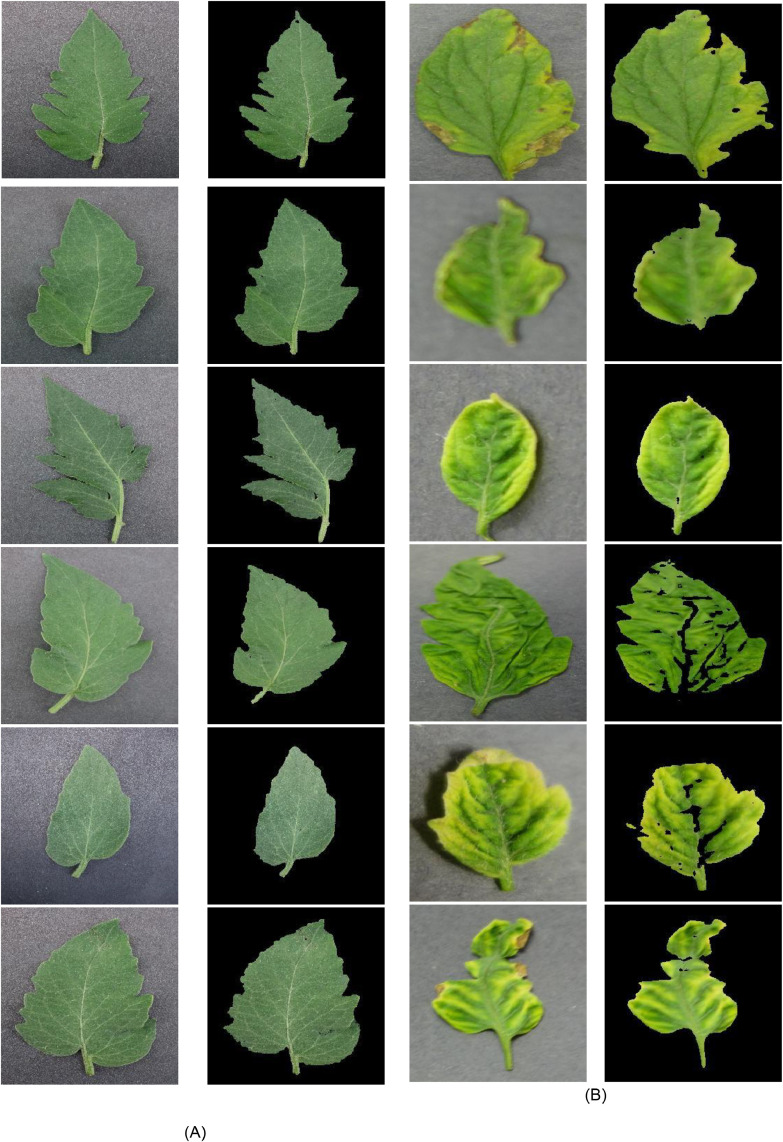
Sample of segmented images, (A) Healthy, and (B) infected images.

In image preprocessing, the images of the tomato leaves dataset are segmented to extract the leaf part from the image by suppressing the background pixels. In this study, a threshold-based segmentation method is utilized, in which green and brown masks are individually created with their respective lower and upper threshold values. These threshold values are set based on the background in the image. The output of the Image Segmentation step is shown in Fig. 3. The background segmentation and the use of the YCbCr color space can significantly enhance the classification of tomato leaves. By segmenting the background, the tomato leaves isolated from the irrelevant parts of the image, ensuring that the proposed feature extraction model focuses on the features that matter most.The background segmentation ensures a consistent background across all images, which helps the model to generalize better and reduces overfitting to specific background patterns.

## Color spaces conversion

The original images were converted to the YCbCr color space from the RGB color space, which is made up of the red, blue, and green components. This representation of the YCbCr color space model is independent of the device that uses a human-vision-based color representation scheme. The choice of color space components for tomato leaves classification depends on various factors such as the specific characteristics of the leaves, the imaging conditions, and the classification algorithm being used. The YCbCr color space can be a suitable choice for leaf classification tasks due to its ability to separate luminance (Y) from chrominance (Cb and Cr) components. The YCbCr color space separation helps in focusing on color information (Cb and Cr) without being affected by lighting conditions, which is critical for color-based classification tasks. Since the chrominance channels are less affected by changes in lighting, the classification becomes more robust to variations in illumination.Moreover,By transforming RGB into YCbCr, redundancy in the color information is reduced, making the classification process more efficient.

## Proposed features extraction

Image texture is a critical component in many image analyses, and tomato leaves image classification greatly benefits from the new handcrafted feature extraction based on conformable polynomials (CP) extraction method. Polynomial-based feature extraction techniques are the most significant since they can detect minor variations in the intensity values of an image. A class of polynomials known as conformable polynomials can be made to fit datasets or functions, providing flexibility and effectiveness in a range of applications, such as image processing [22,23]. There are a few steps involved in using conformable polynomials for feature extraction from images, but they are especially helpful because they can be adjusted to fit various scales and patterns in image data. Prior to using conformable polynomials, preprocessing of the image is required. To lower computational complexity, this can involve color conversion and background segmentation. The suggested conformable polynomial is used to approximate each pixel in the image. Different features can be extracted from the polynomial coefficients and the structure of the fitted polynomials. The polynomial term coefficients can be used as features to represent local intensity variations. A feature vector that represents the image is created by combining the extracted features. One application for this feature vector is image classification. The benefit of using conformable polynomials is that they can be adjusted to match a broad range of picture structures. Additionally, they can offer a condensed depiction of the features of an image. Robust and discriminative features can be extracted from images, improving the performance of subsequent image processing tasks, by utilizing the flexibility and efficiency of conformable polynomials.

The proposed CP feature extraction model is created to extract the texture from tomato images. The goal of proposing the structural features of images with polynomial representations is the motivation behind the use of CPs for image feature extraction. The CPs model draws its inspiration from conformable calculus, which has been thoroughly investigated in various mathematical applications. Image feature extraction which is based on polynomials can identify subtle variations in image intensity values [24]. Polynomial plays a significant role in image processing because of its characteristics for determining pixels and edges in the image. Every image can be recognized by a polynomial in two variables [25,26]. The Tutte polynomial is a perfect model for feature extraction in this study because of its extensive and well-developed theory. Because it is specified for two variables, the Tutte polynomial indicates a substantial amount of information about the image, and its formula is given by the following construction [27]:

(1) $$T\left(X,Y\right)=\;\sum_{i,j}w_{i,j}X^{i}\;Y^{j},$$

where w_i,j_ indicates the connections (weight values) and Xi (training targets at the position i) and Yj (prediction values at the position j ).

The conformable calculus (CC) indicates the following construction [28,29]: Suppose that ℓ is the fractional (arbitrary) value power such that ℓ ϵ [0, 1]. The operator $\delta^{l}$ is considered as a conformable differential if $\delta^{0}$ is the self-operator and $\delta^{1}$ is the traditional difference operative. Absolutely, $\delta^{l}$ is conformable only if differentiable utility ϕ =ϕ $\left(x\right),\;\delta^{0}\;\phi \left(x\right)=\phi \left(x\right),\;\delta^{1}\phi \left(x\right)=\phi^{\prime }\;\left(x\right).$ Anderson and Ulness [28] recently introduced a novel CC preparation influenced by control theory. The following is associated with the definition.

Definition 1 Assume that ℓ ϵ [0, 1], then CC has in the subsequent documented.

(2) $$\;\delta^{l}\phi \left(x\right)=\;\mu_{1}\left(l,x\right)\phi \left(x\right)+\mu_{0}\;\left(l,x\right)\;\phi^{\prime }\left(x\right)$$

where $\delta^{l}\phi \left(x\right)$ is called the conformable differential for the functionϕ(x) with

$\mu_{1}$ and $\mu_{0}$ attain the boundaries.

$$\lim_{\ell \;\to 0}\mu_{1}\left(l,x\right)=1,\;\lim_{\ell \;\to 1}\mu_{1}\left(l,x\right)=0,$$

$$\lim_{l\to 0}\mu_{0}\left(l,x\right)=0,\;\lim_{\ell \;\to 1}\mu_{0}\left(l,x\right)=1.$$

To reach the above explanation, we shall measure both.

(3) $$\;\mu_{1}\left(l,x\right)=\frac{\left(1-l\right)}{\Gamma \left(1+l\right)}x^{l}\text{and}\mu_{0}\left(l,x\right)=\frac{l}{\Gamma \left(1+l\right)}x^{1-l}$$

Thus, the fractional tuning connections between the function ϕ and its derivative are represented by the $\mu_{1},\mu_{0}\;.$

Asum T(X,Y) represents the input image. Now, by operating (1) in (2), we have CTPs as follows:

(4) $$\;{\delta^{l}}_{X}T\left(X,Y\right)=\;\mu_{1}\left(l,h\right)\;T\left(X,Y\right)+\mu_{0}\;\left(l,h\right)\;T^{\prime }\left(X,Y\right)$$

The input image is represented by T(X,Y), while $T^{\prime }\left(X,Y\right)\;$represents the image derivative. The image derivative is a comprehensive representation of the image gradient capturing different aspects of image structure. By substitute the values of both μ0 and μ1 in (3), the following is the proposed texture features expression:

(5) $$\;{\delta^{l}}_{Y}T\left(X,Y\right)=\;\frac{\left(1-l\right)}{\Gamma \left(1+l\right)}h^{l}\;T\left(X,Y\right)+\frac{l}{\Gamma \left(1+l\right)}\;h^{1-l}\;T^{\prime }\left(X,Y\right)$$

**Fig. 4:**
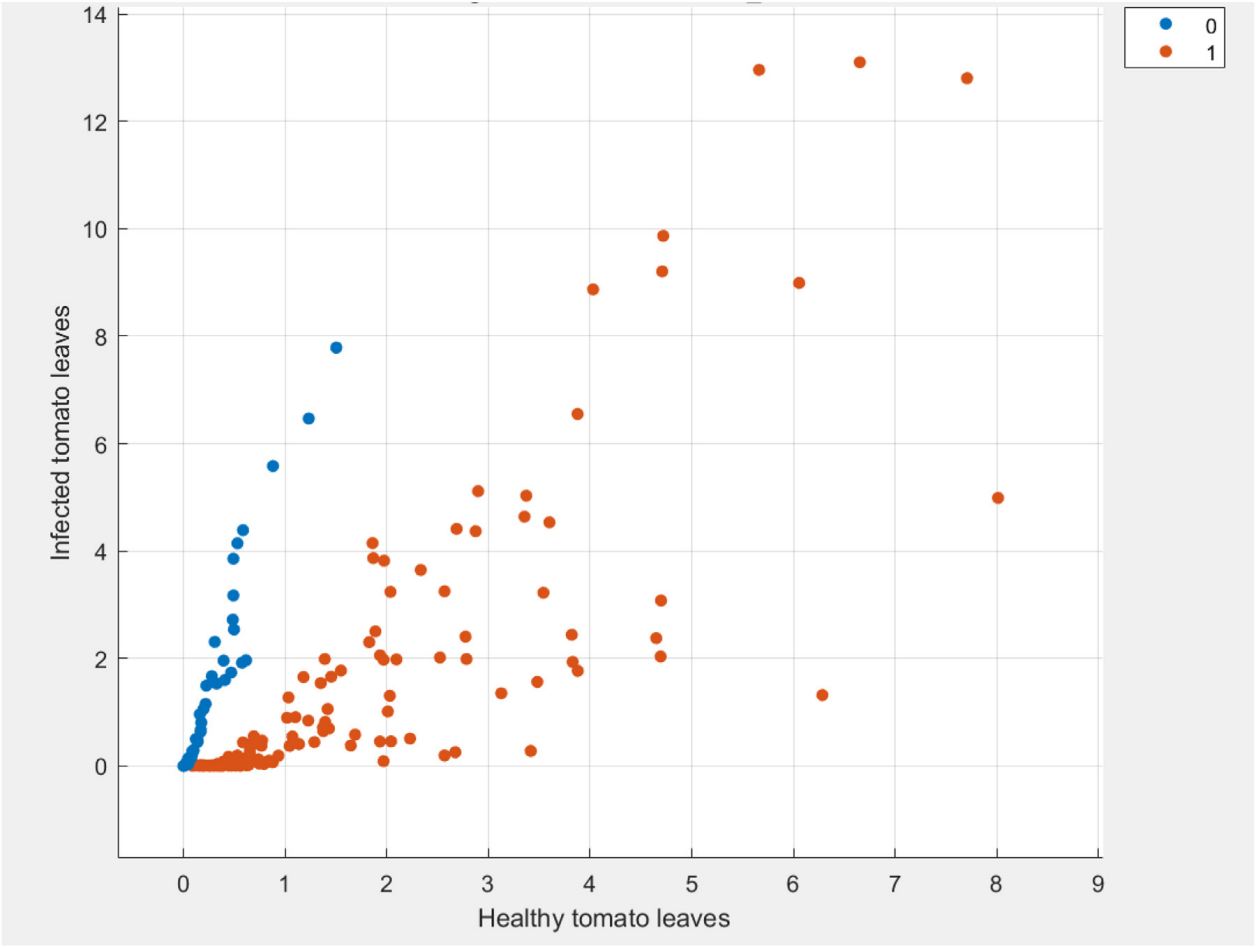
The distribution of the two classes features (tomato leaves as healthy (blue dots) or infected (red dots)).

The proposed texture feature is constructed by two parts: the enhanced based term $\frac{\left(1-l\right)}{\Gamma (1+l)}h^{l}\;T\left(X,Y\right)$, and the texture detail part $\frac{l}{\Gamma (1+l)}\;h^{1-l}\;T^{\prime }\left(X,Y\right)$ where T is the input image, h denotes pixel probability, and l is the fractional conformable number. In an experiment, the fractional conformable number was set to 1.5, which produced the best texture characteristics. The input image T(X,Y) is first divided into non-overlapping blocks for the feature extraction. As seen in Fig. 4, the feature distribution provides a quantitative basis for separating images that are healthy from those that are infected which proves that the proposed CPs is a method for extracting distinctive features from tomato leaves images.

In order to save computational costs and resource allocation, the dimensionality reduction of features is applied after the suggested texture features expression is extracted from each image using Eq (5). This ensures that the algorithm is operating at its most optimal and efficient settings. In the current work, the proposed feature extraction model uses the measures of "Mean", "Variance", "Skewness", and "Kurtosis" to reduce the dimensionality of the extracted data in each image.

For features F of M scalar observations, the “Mean” is defined as

(6) $$Mn=\frac{1}{M}\sum_{I=1}^{M}F_{i}$$

The definition of a "Variance" is

(7) $$Vc=\frac{1}{M-1}\sum_{i=1}^{NM}{\left|F_{i}-\mu \right|}^{2}$$

where μ is the “Mean” of F_i_

The asymmetry of the feature data around the feature mean is measured as a quantity called "skewness," which is defined as

(8) $$Ss=\frac{V{\left(x-\mu \right)}^{3}}{\sigma^{3}}$$

where V(t) represents the estimated value of the quantity t and σ is the "Standard deviation." "Kurtosis" is described as

(9) $$Ks=\frac{V{\left(x-\mu \right)}^{4}}{\sigma^{4}}$$

The ‘Standard deviation’ is described as

(10) $$St=\sqrt{\frac{1}{M-1}}\sum_{i=1}^{M}{\left|F_{i}-\mu \right|}^{2}$$

## Classification

This study compares the accuracy, precision, recall, and F1 classification of tomato leaf infections for KNN, SVM, and logistic regression classifiers. In machine learning techniques, the KNN algorithm is a straightforward classifier that performs excellently for simple recognition problems. Based on the sample's distance from its closest k sample points, KNN predicts which class a new sample will belong to when it is to be tested. For binary classification issues, the supervised learning algorithm logistic regression is employed. It is extensively utilized in many industries, including marketing, finance, and healthcare. The logistic function, sometimes referred to as the sigmoid function, is used by the logistic regression model to simulate the likelihood that an input will belong to the positive class. The logistic regression classifier is trained by determining the best parameters to minimize the cross-entropy loss function, or cost function. The SVM algorithm is a machine learning algorithm that resolves binary classification issues by applying statistical learning theory.

## Evaluation metrics

The classification results were evaluated by the accuracy, precision, recall, and F1 score as follows:

(11) $$\text{Accuracy}\;=\frac{\text{TP}\;+\;\text{TN}}{\text{TP}\;+\;\text{FP}\;+\;\text{TN}\;+\;\text{FN}}$$

(12) $$\text{Precision}\;=\frac{\text{TP}}{\text{TP}\;+\;\text{FP}}$$

(13) $$\text{Recall}\;=\frac{\text{TP}}{\text{TP}\;+\;\text{FN}}$$

(14) $$F1\;=\frac{2\text{PR}}{P\;+\;R}$$

where the correctly predicted positive values are called true positive (TP), the incorrectly predicted positive values are called false positive (FP), the correctly predicted negative values are called true negative (TN), and the incorrectly predicted negative values are called false negative (FN).

The three most important metrics for assessing tomato leaf disease classification models are precision, recall, and F1 score.. Recall ensures comprehensive detection, Precision ensures accurate identification of diseased plants, and the F1 score sF1 score balances both aspects to provide a reliable measure of overall performance. For the purpose of controlling tomato leaf diseases in agricultural settings, these metrics are essential for creating reliable, dependable, and efficient systems.

When evaluating the performance of an image classification model, it's important to quantify its accuracy and understand the variability of its performance. Here's a breakdown of how to compute the mean accuracy, standard deviation, and confidence intervals:

1. Mean Accuracy

Mean accuracy is the average accuracy of the model over multiple runs or folds in cross-validation. It provides a single metric to summarize the performance of the model.

2. Standard Deviation

The standard deviation calculates how far the accuracy scores deviate from the mean accuracy. A low standard deviation indicates that the accuracy scores are close to the mean, whereas a high standard deviation indicates a wide range of accuracy scores.

3. Confidence Intervals

Confidence intervals provide a range within which we can expect the true accuracy of the model to lie with a certain level of confidence (typically 95 %).

## Method validation

MATLAB 2021b was utilized for the execution of each test. The process of validation of the feature extraction has been achieved by splitting the complete image dataset into three sections: testing, validation, and training. This has enhanced the model's capacity to generalize and prevented overfitting. Plus. This study uses the 5-fold cross-validation technique. The machine learning algorithm's performance on a dataset is evaluated using nested 5-fold cross-validation, which divides the data into 5 equal-sized folds, 4 of which are used for training and 1 for testing. In this five-fold process, each fold is tested once, and the four folds that remain are used for training. By minimizing the potential for overfitting to the training set, the nested 5-fold cross-validation technique enables a more precise assessment of the machine learning algorithm's performance.

**Fig. 5:**
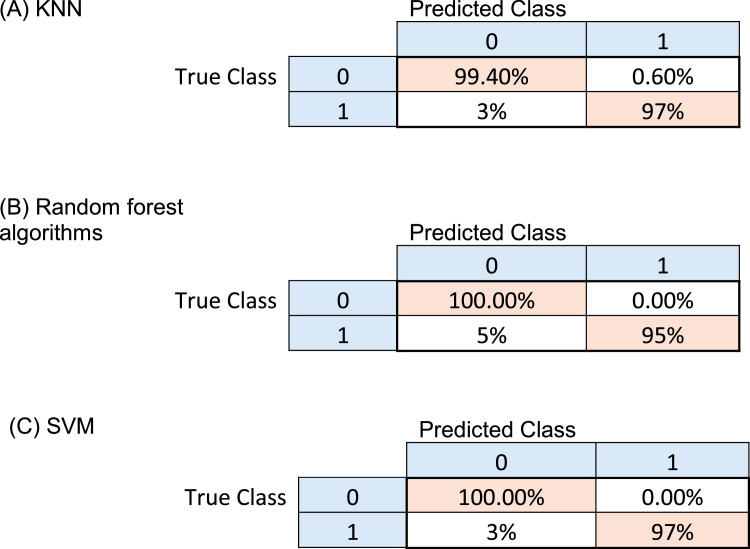
Confusion matrix for healthy, and unhealthy tomato leaf image classification.

Three distinct classifiers are trained using the features retrieved by the suggested CPs method in machine learning for the classification of tomato infections, with the goal being to identify which classifier performs the best in terms of classification. Fig. 5 shows the confusion matrices for the algorithms, and the SVM classifiers were the best, followed by the KNN algorithm. KNN and SVM, classifiers. This demonstrates that the quality of the extracted features had a direct impact on the final classification result in the classification task.

The tested machine learning techniques had accuracy levels of 98.80 % (for random forest algorithms), 98.50 % (KNN), and 98.80 % (SVM). The random forest algorithms and SVM classifiers produced the best classification results out of the three machine learning techniques that were tested; the KNN algorithm came in second.

**Fig. 6:**
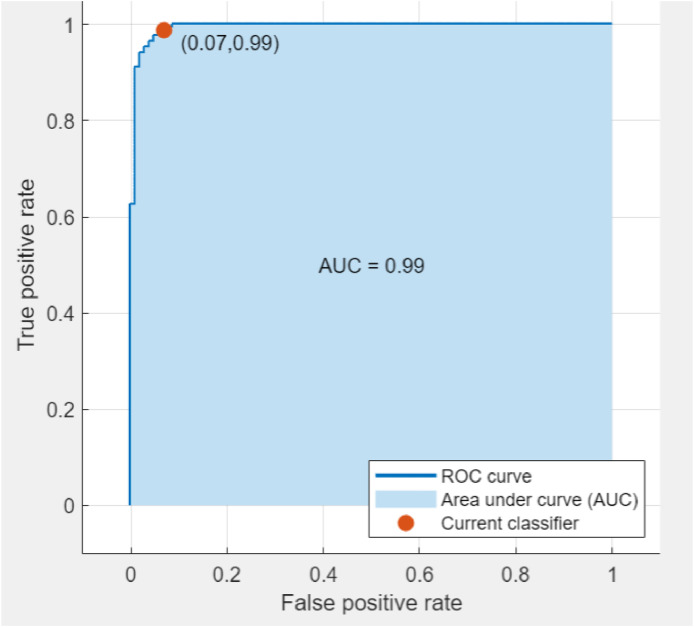
The tomato classification area under the curves (AUCs).

In the classification task, four evaluation indexes were employed: Accuracy, Precision, Recall, and F1. These indices were employed to assess the predictive power of the suggested method. The detailed results are shown in Table 1, where the suggested method yielded an accuracy of 98.80 % for the SVM classifier. Another technique for verifying classification results is the "area under the curve" (AUC) and the "receiver operating characteristic curve" (ROC). The proposed model's ROC curve is shown in Fig. 6. Since the AUC is 0.99 (higher is better), it can be concluded that the categorization classes are more effectively divided.

**Table 1:** The classification results by the KNN algorithm, Random forest algorithms and SVM, classifiers.

	Mean Accuracy %	Standard Deviation	Confidence Intervals	Precision %	Recall %	F1 %
KNN	98.20	0.7071	97.65–98.74	99.40	97.07	98.22
Random forest algorithms	97.50	0.5962	97.07–97.92	100	95.24	97.56
SVM	98.80	0.8165	98.21–99.38	100	97.09	98.52

To illustrate the efficacy of the proposed feature extraction approach, a comparison made by previous studies which investigated the classification of tomato leaves to improve the differentiation between healthy and infected cases. Table 1 shows the performance comparison to another research that applied same image dataset (PlantVillage tomato dataset). The following existing methods which were developed for tomato leaf disease classification.

Tan et al. [1], method performed using 52 texture features for tomato leaf diseases classification. This study applied both the machine learning and deep learning networks.

Badiger method [17] developed a leaf disease classifier using SVM. The authors standardized the image sizes and applied k-means clustering for image segmentation.

In the study proposed by Sabrol and Satish [15], texture, shape, and color features were extracted from images of tomato plants. The segmentation process is completed before moving on to the feature extraction step. The classification algorithm and fusion of multiple features for identifying and categorizing tomato leaf disease were introduced by Basavaiah and Arlene Anthony [16].

The study by Lubis et al. [18] proposed the K-NN algorithm and feature extraction for the classification of tomato leaf disease images.

A deep neural network model for identifying and categorizing tomato plant leaf diseases into predetermined groups was studied by Trivedi et al. [11]. It also considered the plant's morphological characteristics, including texture and color.

Improving the overall accuracy of the tomato leaf disease classification is the main goal of this study. According to the finding in Table 1, the proposed CPs feature extraction model improved classification accuracy when compared to other methods.

To illustrate the efficacy of the suggested approach, a comparison made by previous studies which investigated the tomato leaf disease classification using PlantVillage tomato dataset. The "Accuracy" evaluation measure was used in Table 2, and it is the only measure that was shared by all of the studies in Table 2. The study in [11] slightly approaching the proposed SVM classification by 0.31 % in terms of classification accuracy. For the purpose of classifying tomato plant leaf diseases, the study by Trivedi et al. [11] employed a deep neural network model, which increased classification accuracy but required more computing power. Throughout the entire experiment, Google Colab was used. The targeted area of the images was first separated from the original images through preprocessing of the input images. Subsequently, the images undergo additional processing using different CNN model hyper-parameters. Lastly, CNN extracts additional features from images, such as edges, textures, and colors. The CNN classification model in this study contains a large number of complexly correlated parameters, which increases the likelihood of data overfitting during the training phase. Although the suggested model used the fewest feature dimensions to achieve an acceptable classification accuracy. Additionally, the study [15], among other methods, achieved the second good accuracy. This is because the study extracted multiple features from images of healthy and unhealthy tomato plants, including color, shape, and texture features, which improved the classification accuracy. The results demonstrate that the proposed approach uses the fewest feature dimensions while achieving an acceptable classification accuracy. This demonstrates the proposed feature extraction method is useful and reliable as a texture descriptor for image classification.

**Table 2:** Performance comparison on PlantVillage tomato dataset.

Method	Classifiers	Accuracy%
Tan et al. [1]	CNN and SVM	91.00
Badiger et al. [17]	K-Means and SVM	96
Sabrol and Satish [15]	Classification tree	97.30
Basavaiah and Arlene Anthony [16]	Random forest classifier	94
Lubis et al. [18]	KNN	96.58
Trivedi et al. [11]	CNN	98.49
Proposed method	SVM	98.80

Improved crop yields result from the early detection of diseases and prevention of their spread across fields through the use of computer vision techniques. It's critical to identify and classify diseases appropriately in order to prevent unanticipated events. In this study, a new feature extraction method was proposed based on CPs to extract most importance feature from tomato leaves for classification of tomato infections using three machine learning algorithms KNN, Random forest algorithms, and SVM. Through comparative experiments, among all the classifiers, the SVM obtained the best classification results. The method described in this study may also be applied to the identification and categorization of plant disease images. The training models' complexity has decreased, and high classification accuracy has been attained with the proposed feature extraction method. In addition, the outcomes demonstrated the superiority of the proposed feature extraction's experimental performance in contrast to other past studies on tomato leaf diseases. In future work, a multi-infection types will be study, which will be of more practical value.

## Limitations

The proposed method for classification of tomato infections has a limitation in that it can be influenced by artifacts on tomato leaf images, which could cause misclassification because certain diseases have symptoms that are similar on the leaves.

## Ethics statements

This article does not contain any studies with human or animal participants.

## Funding

This research received no external funding.

## CRediT authorship contribution statement

**Ala'a R. Al-Shamasneh:** Methodology, Software. **Rabha W. Ibrahim:** Writing – original draft.

## Declaration of competing interest

The authors declare that they have no known competing financial interests or personal relationships that could have appeared to influence the work reported in this paper.

## Data Availability

Data will be made available on request.
